# Amputation

**Published:** 1856-06

**Authors:** Asa Morgan

**Affiliations:** Dewitt, Iowa


### A CASE OF AMPUTATION---CHLOROFORM ADMINISTERED TO THE PATIENT IN A STATE OF INTOXICATION.

BY ASA MORGAN, M. D., DEWITT, IOWA.

A German boy aged 15 years, living some twelve miles from this place, accidentally shot himself through the right arm, about two inches above the wrist joint, mangling it in a horrible manner and almost completely severing it. It happened on Sat. P. M., Feb. 2. A Homœopath, practicing in the neighborhood, was immediately called in, and upon examining the wound, told the friends that he could do nothing for the case. Immediately a message was sent to De Witt, and Dr. Vary and myself summoned to the case. We arrived early next morning, and found the patient in a condition commonly called "half drunk." The friends had been giving him brandy freely during the night, in order to subdue the pain. On examining the wound, we deemed it necessary to amputate at once, and immediately made ready for the operation. Dr. Vary administered the chloroform, whilst the patient was still in a state of intoxication, with the happiest effect. I proceeded to amputate the limb, and during the whole operation the patient did not show any signs of pain, but seemed to be in natural sleep, with the countenance unchanged from that of health.

The pulse remained good and did not sink as it generally does, after the administration of chloroform. In about 8 minutes from the time the anæesthetic first produced its effect, the patient awoke as if from a natural sleep. The effects passed off without the amount of exhaustion usually attending the administration of chloroform. I believe that the depressing effects of the chloroform on the patient were entirely prevented by the timely use of the alcohol; by producing more of a permanent stimulus on the system, and thereby enabling it to bear up better under the effects of the chloroform, as well as the operation.

I believe that chloroform may be advantageously administered in cases of extreme debility, where it is necessary to perform a surgical operation, by giving brandy freely some hours previously to the operation. The anæsthesia will be as perfect, and the depressing effects of the chloroform less dangerous, by first getting up a gradual stimulus before attempting to operate. I shall make farther trial of this mode of administering chloroform in the future, to all cases applicable, and report the success. The patient above mentioned is at this time doing well.

